# New Delivery Systems for Local Anaesthetics—Part 2

**DOI:** 10.1155/2012/289373

**Published:** 2011-12-08

**Authors:** Edward A. Shipton

**Affiliations:** Department of Anaesthesia, University of Otago, Christchurch 8042, New Zealand

## Abstract

Part 2 of this paper deals with the techniques for drug delivery of topical and injectable local anaesthetics. The various routes of local anaesthetic delivery (epidural, peripheral, wound catheters, intra-nasal, intra-vesical, intra-articular, intra-osseous) are explored. To enhance transdermal local anaesthetic permeation, additional methods to the use of an eutectic mixture of local anaesthetics and the use of controlled heat can be used. These methods include iontophoresis, electroporation, sonophoresis, and magnetophoresis. The potential clinical uses of topical local anaesthetics are elucidated. Iontophoresis, the active transportation of a drug into the skin using a constant low-voltage direct current is discussed. It is desirable to prolong local anaesthetic blockade by extending its sensory component only. The optimal release and safety of the encapsulated local anaesthetic agents still need to be determined. The use of different delivery systems should provide the clinician with both an extended range and choice in the degree of prolongation of action of each agent.

## 1. Introduction

A drug delivery system should have minimal tissue reaction, a reliable drug release profile, and well-defined degradation rate for biodegradable carrier until all nontoxic products are excreted [1]. For local anaesthetics (LAs), the development of new effective delivery systems intends to suitably modulate the release rate of these drugs, extend their anaesthetic effect, and enhance their localisation; this reduces problems of systemic toxicity. Part 2 of this paper deals with the innovations pertaining to formulations, and techniques for drug delivery of topical and injectable local anaesthetics.

## 2. Routes of Local Anaesthetic Delivery

### 2.1. Epidural

Patient controlled epidural analgesia (PCEA) allows patients to self-administer drug doses according to their analgesic needs. This route relies on a staff-programmed pump and skilled and qualified members of the hospital staff for administration. Both local anaesthetics and opioids are agents for epidural analgesia. The use of epidural local anaesthetics is associated with a higher incidence of hypotension, motor block, and urinary retention, compared with use of opioids [2]. However, in a recent meta-analysis, only a continuous infusion of epidural local anaesthetics was superior to intravenous opioids in improving pain control and reducing adverse effects [3].

### 2.2. Peripheral

**Figure 1:**
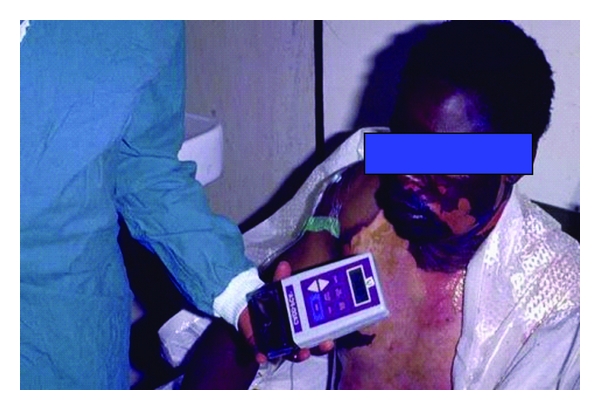
Pharmacia Deltec CADD Ambulatory Infusion Pump.

**Figure 2:**
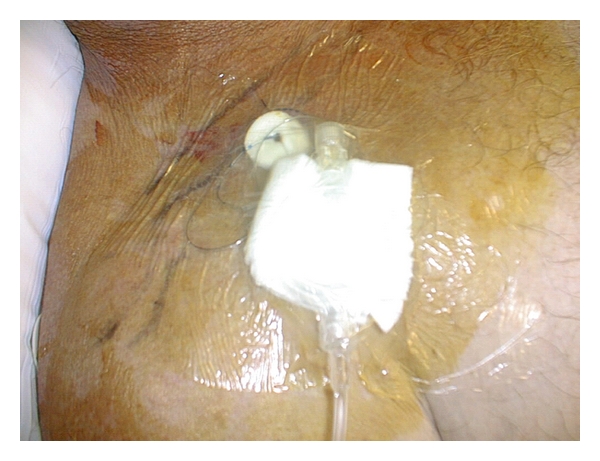
Patient-controlled perineural analgesia via infraclavicular brachial plexus catheter.

Patient-controlled regional analgesia (PCRA) encompasses a variety of techniques that provide effective postoperative pain relief without systemic exposure to opioids. Using PCRA, patients initiate the delivery of small doses of local anaesthetics (ropivacaine, bupivacaine) via an indwelling catheter that can be placed in different regions of the body depending upon the type of surgery. In some cases, a combination of local anaesthetic and opioid is administered. Infusions are controlled either by a staff-programmed electronic pump (Figure 1) or by a disposable elastomeric pump [3]. An elastomeric pump is a device that has a distensible bulb inside a protective bulb with a built-in filling port, delivery tube, and an antibacterial filter. Antibacterial filters are recommended with blocks involving a nerve plexus (and in neuraxial blocks). Analgesia can be delivered directly into a surgical incision (incisional PCRA), the intra-articular tissue (IA PCRA), or the perineural site (perineural PCRA) (Figure 2) [2].

### 2.3. Wound Catheters

**Figure 3:**
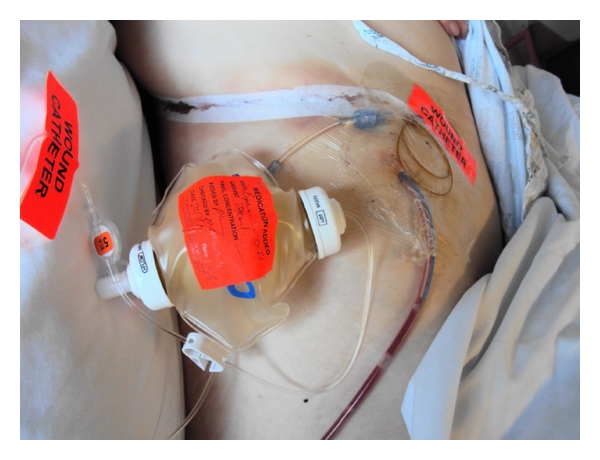
Elastomeric pump for a wound catheter.

The insertion of wound catheters allows for continuous infusions of local anaesthetics into the surgical wound at the end of the procedure (Figure 3). Continuous wound catheters can confer several benefits, including improved analgesia, reduced opioid use and adverse effects, increased patient satisfaction, and reduced hospital stay [4].

The use of continuous wound catheters consistently reduces the need for opioids (both rescue and total dose). Patients have consistently rated postoperative nausea and vomiting (PONV) as a primary concern after surgery [5, 6]. The reduced need for opioids (though an infrequently measured result in Randomised Controlled Trials) might contribute to increased patient satisfaction [4]. Reduced length of hospital stay has been associated with continuous wound catheters, especially in the cardiothoracic and orthopaedic surgery subgroups [4]. Meta-analysis suggested the potential saving of one day of hospital stay [4]. Incidences of technical failure and local anaesthetic toxicity from wound catheters are low. Several reports have raised potential concern about wound infections from the presence of a catheter [7]. However, reported wound infection rates were found to be similar between active (0.7%) and control groups (1.2%) [4].

### 2.4. Intranasal Route

The nasal mucosa acts as an anatomical obstacle hard to get over, except for compounds with low molecular weight or highly lipophilic compounds. For the manipulation of nasal fractures, lignocaine and cocaine have been used in topical local anaesthesia in the form of a spray, paste, or on cotton wool and pledgets; this appears to be a safe and effective alternative to general anaesthesia. A systematic review has found no significant differences between local anaesthesia and general anaesthesia as regards pain, cosmesis or nasal patency after nasal fracture manipulation performed under topical local anaesthesia [8]. A recent Cochrane review showed that nasopharyngeal topical local anaesthetic or vasoconstrictor preparations prior to the use of a fibre-optic nasal endoscope did not demonstrate any advantages in using a topical treatment prior to endoscopy [9]. In contrast, the very lipophilic opioids, such as fentanyl are rapidly absorbed via intranasal administration (with a bioavailability close to 90% for fentanyl) and are used for the treatment of breakthrough pain [10].

### 2.5. Intravesical (Bladder) Route

**Figure 4:**
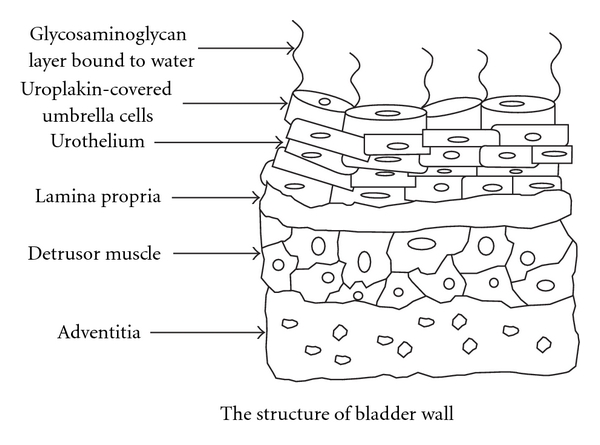
The structure of the urinary bladder wall and urothelium.

The transport of local anaesthetics through the urothelium into deeper layers of the bladder has been significantly enhanced by electromotive drug administration (Figure 4) [11]. It is a cost-effective way to deliver lignocaine with greatly improved penetration rate into the bladder wall [12]. Vehicles such as electromotive drug administration, new in situ delivery systems, and bioadhesive liposomes make it possible to extend intravesical therapy and drug administration to many bladder diseases [11].

### 2.6. Intra-Articular (IA) Route

For microspheres destined for intra-articular delivery, the following properties have been identified as important, namely, size (within the range of 4-5 *μ*m in diameter), resistance to in vivo degradation, minimal leakage of drug, and biocompatibility [13]. Prolonging the intra-articular drug residence time of LAs may be met by formulating microspheres that have been rationally designed to optimise uptake by the synovium [13]. However, the causes of 23 cases of postarthroscopic glenohumeral chondrolysis were recently analysed. Several common factors were found, but no single mechanism was shown. Multiple factors seemed to be implicated. The use of intra-articular local anaesthetics is probably not advisable until the role of local anaesthetics in this regard has been clarified, as chondrolysis has been the subject of many lawsuits [14].

### 2.7. Intra-Osseous Route

Computer-controlled local anaesthetic delivery devices (C-CLAD) and systems for intra-osseous injection are important additions to the dental anaesthesia armamentarium [15]. C-CLAD using slow infusion rates can significantly reduce the discomfort of local anaesthetic infusions, especially in palatal tissues; C-CLAD facilitates palatal approaches to pulpal nerve block. It should find special use in cosmetic dentistry, periodontal therapy, and paediatric dentistry [15].

## 3. Transdermal Route of Local Anaesthetic Drug Delivery

**Figure 5:**
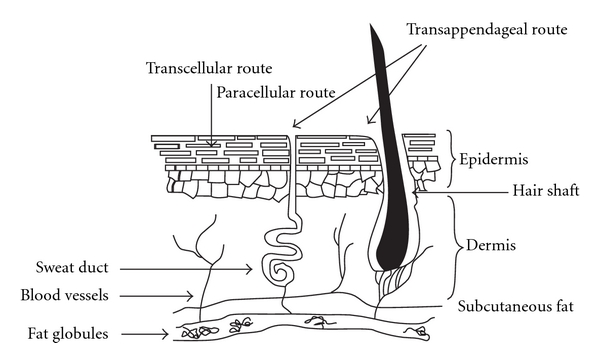
Transdermal routes of absorption of local anaesthetics.

Transdermal drug delivery is one of the most patient-compliant routes of drug administration. However, the stratum corneum, the outer most layer of the skin resists the penetration of drugs across the skin (Figure 5). Hydrophilic, ionised, and macromolecular substances are poorly permeable across the skin [16]. To enhance drug permeation in a passive manner, transdermal drugs should be lipophilic and should ideally have a molecular weight less than 500 Daltons [17]. Alternatively, energy-dependent active measures can be used to enhance drug delivery across the skin. These include physical permeabilisation of skin or by driving the drug molecule across the skin. In addition to the use of an eutectic mixture of local anaesthetics, and the use of controlled heat, other methods such as iontophoresis, electroporation, sonophoresis, and magnetophoresis can be used [16].

### 3.1. Magnetophoresis

**Figure 6:**
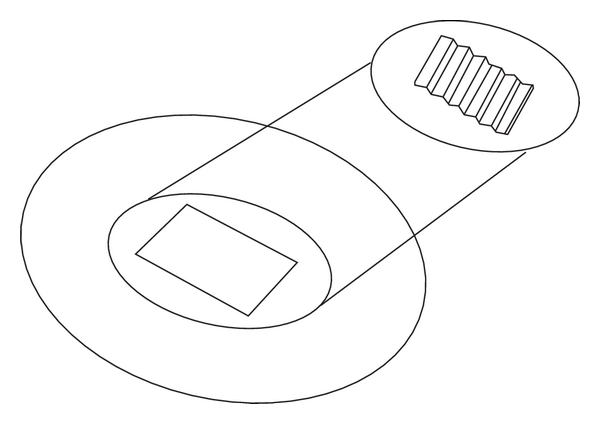
Magnetophoresis.

Magnetophoresis is a method of enhancement of drug permeation across the biological barriers by the application of a magnetic field (Figure 6). The predominant mechanism responsible for magnetically mediated drug permeation enhancement is known as “magnetokinesis” [17]. The octanol/water partition coefficient of drugs increases when exposed to the magnetic field [17]. Magnetophoretic patch systems deliver drugs at a higher rate than nonmagnetic patch systems. In the rat model, the dermal bioavailability (Area Under the Curve 0–6 hours) from the magnetophoretic patch system was significantly higher compared to a non-magnetic control patch [17].

### 3.2. Sonophoresis/Phonophoresis

**Figure 7:**
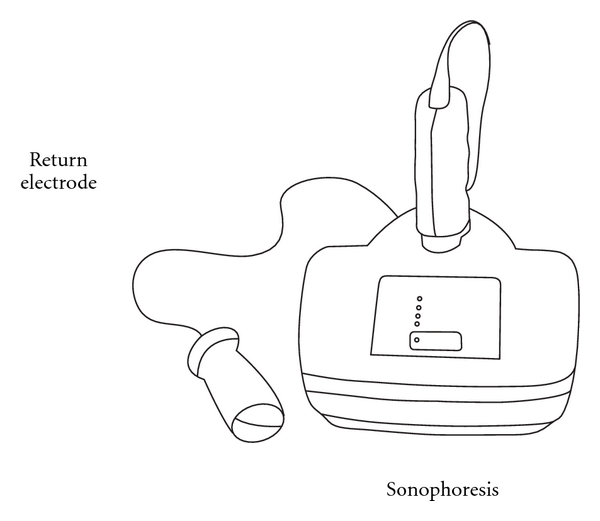
Sonophoresis—an ultrasonic skin permeation device.

The use of ultrasound for the delivery of drugs to, or through, the skin is commonly known as sonophoresis or phonophoresis (Figure 7). The frequency of the ultrasound wave corresponds to the number of times that the transducer tip is displaced per second of application time. High-frequency sonophoresis includes frequencies in the range of 0.7–16 MHz (most commonly 1–3 MHz) [18]. Low-frequency sonophoresis includes frequencies in the range of 20–100 kHz and allows transdermal delivery of both hydrophilic and high-molecular mass permeants at therapeutic levels [19].

The main contributor responsible for skin permeability enhancement by sonophoresis is acoustic cavitation. This causes the formation of acoustic microjets on the surface of the skin in a nonuniform manner [19]. When surfactant is included in the treatment of skin with low-frequency sonophoresis, a strong synergistic enhancement in skin permeability occurs, allowing delivery of hydrophilic permeants. Additionally, low-frequency sonophoresis-mediated transdermal delivery can be used to deliver macromolecules, including liposomes and nanoparticles [19]. This technology is currently approved for use by the Federal Drug Administration (FDA) for local anaesthetics. In volunteers, a transducer has been used to administer an anaesthetic drug transdermally. When 0.5 MHz ultrasound in phonophoresis used for conduction anaesthesia with lignocaine hydrochloride, it was found to be more effective than the 1 MHz widely used in clinical situations (Figure 7) [20].

### 3.3. Microporation Technologies

Skin microporation may be considered a minimally invasive technology that can be broadly divided into microneedle technology, thermal microporation, and laser ablation [21]. To improve the rate and extent of transdermal lignocaine across porcine ear skin, the application of novel laser microporation technology (Painless Laser Epidermal System) has been used to create well-defined conduits in the skin [22].

### 3.4. Electroporation

Electroporation is the application of short high voltage pulses that result in formation of transient aqueous pathways for diffusion of molecules across the skin [16]. In case of electroporation, the electrical pulses are applied only for fraction of a second; the interval between subsequent pulses allows the skin to depolarise [16]. Therefore, polarisation of skin does not interfere with the current flow or drug diffusion. In the porcine epidermis, the transport of lignocaine hydrochloride in case of low voltage electropulsation was found to be 8-fold more than the control [16]. The amount of lignocaine hydrochloride present in the epidermis was found to be 2-fold higher as well.

Electrokinesis (electrophoresis and or electro-osmosis) and permeabilisation of membranes are responsible for enhanced transdermal drug transport by low voltage electroporation. The total duration of electrical current application during 20 minutes of low voltage electroporation is only one minute. Low voltage electroporation enhances drug permeation relatively more efficiently than constant direct current iontophoresis [16].

### 3.5. Eutectic Patches

A new topical local anaesthetic eutectic patch consisting of a mixture of lignocaine 70 mg and tetracaine 70 mg (Synera in the United States, Rapydan in Europe) has been developed [23]. This patch has an integrated heating element intended to enhance the flux of the tetracaine and lignocaine leading to more rapid and effective delivery of the local anaesthetics to the target area. The patch starts heating once removed from the pouch and exposed to atmospheric oxygen; it may increase skin temperature by up to 5°C [23].

To study the use of lignocaine/tetracaine-medicated plasters in patients undergoing minor dermatological or vascular access procedures, patients were randomised in a double-blind clinical trial [24]. Patient-reported median pain scores with the use of lignocaine/tetracaine-medicated plasters were found to be significantly lower than those with an identical plaster-containing placebo [24].

### 3.6. Clinical

With the use of topical local anaesthetics for dermal laceration repair, a meta-analysis reviewed 22 trials with more than 3000 randomised patients; it concluded that topical tetracaine, bupivacaine, and lignocaine had an equivalent or superior analgesic efficacy to the intradermal infiltration of cocaine-containing anaesthetics. The topical preparations proved less expensive and were safer [23, 25]. In another recent meta-analysis of 25 trials with more than 2000 subjects, three topical anaesthetics, (tetracaine, liposome-encapsulated tetracaine, and liposome-encapsulated lignocaine) were found to be equally efficacious [26]. Tetracaine had the added advantage of a longer duration and was reported not to cause methemoglobinemia [27]. Another study has demonstrated that the lignocaine/tetracaine patch provided better local anaesthesia than the control at all application times under 60 minutes [28]. 

Potential uses of topical anaesthetics include the following: for gaining pain-free vascular access; for skin or punch biopsies; for bone marrow aspiration; for treatment of postherpetic neuralgia; for immunisation; for myofascial pain due to trigger points; for nerve entrapment such as carpal tunnel syndrome; for regional block placement; and for dermal procedures requiring laser treatment.

## 4. Iontophoresis

**Figure 8:**
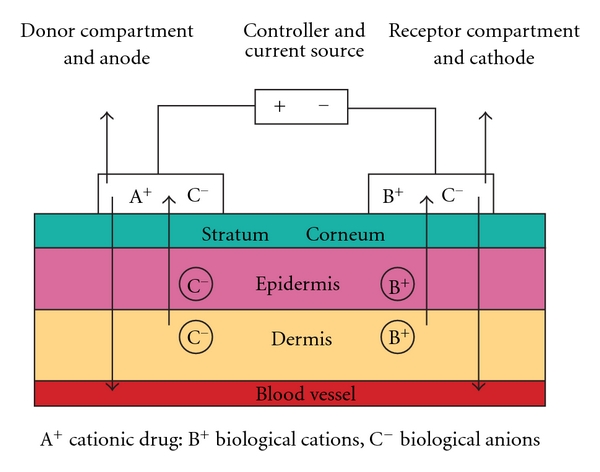
Iontophoresis. Components of an anodal iontophoretic device are a current source; a current control device; anode (donor) reservoir system (with a positively charged drug/ion in solution); cathode reservoir system (on a different skin site). With an electric current, all cations move away from the anode and into the skin, and negatively charged ions move from the body into donor reservoir.

Iontophoresis involves the active transportation of a drug into the skin using a constant low-voltage direct current. Ions migrate between electrodes of opposite charges, promoting ion transport through the skin (Figure 8) [29]. A direct electrical current facilitates the dermal penetration of positively charged lignocaine molecules when placed under a positive electrode for local anaesthesia. Physicochemical properties, such as good aqueous solubility, and the presence of charged groups that render peptides and proteins “difficult to deliver” by other approaches, are ideal for iontophoresis [30] The amount of drug delivered via iontophoresis is dependent on the current and the duration of delivery. The control afforded by constant current iontophoresis over transport rates means that peptide/protein delivery kinetics could mimic endogenous secretion profiles [30]. Moreover, complex input kinetics can be used to optimise and individualise therapy.

Iontophoresis has been known to cause skin irritation at higher current densities or upon longer application [16, 31, 32]. Moreover, when direct current electric field is applied over longer durations, an electrochemical polarisation occurs in the skin which decreases the magnitude of current flow through the skin [16]. This in turn could affect the amount of drug ions driven across the skin.

Small, portable iontophoresis devices have been developed. Dermal anaesthesia can be achieved fairly rapidly using lignocaine iontophoresis without needles [29]. Adrenaline added to the lignocaine solution enhances the effect and duration of local anaesthesia during iontophoresis; this is due to the local vasoconstriction inhibiting lignocaine absorption into the systemic circulation.

Delivery can be hastened by using ultrasound. In a Randomised Controlled Trial, ultrasound pretreatment plus two-minute low-voltage iontophoresis provided better skin anaesthesia than sham-ultrasound plus two-minute low-voltage iontophoresis, and similar to standard, 10-minute high-voltage iontophoresis [33]. Lignocaine HCl 10%/Adrenaline 0.1% topical iontophoretic patch (LidoSite) is the first FDA-approved prefilled active anaesthetic patch. In volunteers, it was found that 2% lidocaine could be delivered up to 5 mm below the surface of the skin when the drug compound contained adrenaline, and when passive delivery occurred for at least 50 minutes after the active delivery has terminated [34].

## 5. Future

It is desirable to acquire the ability to prolong local anaesthetic blockade and, if possible, extend only its sensory component. This paper portrayed the innovative techniques for the drug delivery of topical and injectable local anaesthetics. However, the optimal release and safety of the encapsulated LA agents still need to be determined. Using encapsulation to avoid systemic toxicity, the nonconventional local anaesthetics (tetrodotoxin, saxitoxin) show promising efficacy [1]. Recently, lignocaine-coated microneedles have been developed for rapid, safe, and prolonged local analgesic action [35]. These delivery systems should provide the clinician with both an extended range and a choice in the degree of prolongation of action of each agent [36].
